# Limited phosphorus availability is the Achilles heel of tropical reef corals in a warming ocean

**DOI:** 10.1038/srep31768

**Published:** 2016-08-17

**Authors:** Leïla Ezzat, Jean-François Maguer, Renaud Grover, Christine Ferrier-Pagès

**Affiliations:** 1 Centre Scientifique de Monaco, Marine Department, Principality of Monaco, 8 Quai Antoine Ier, MC-98000, Monaco; 2 LEMAR - UMR 6539 UBO/CNRS/IRD, Institut Universitaire Européen de la Mer, Place Nicolas Copernic, 29280 Plouzané, France

## Abstract

During the 20^th^ century, seawater temperatures have significantly increased, leading to profound alterations in biogeochemical cycles and ecosystem processes. Elevated temperatures have also caused massive bleaching (symbiont/pigment loss) of autotrophic symbioses, such as in coral-dinoflagellate association. As symbionts provide most nutrients to the host, their expulsion during bleaching induces host starvation. However, with the exception of carbon, the nutritional impact of bleaching on corals is still unknown, due to the poorly understood requirements in inorganic nutrients during stress. We therefore assessed the uptake rates of nitrogen and phosphate by five coral species maintained under normal and thermal stress conditions. Our results showed that nitrogen acquisition rates were significantly reduced during thermal stress, while phosphorus uptake rates were significantly increased in most species, suggesting a key role of this nutrient. Additional experiments showed that during thermal stress, phosphorus was required to maintain symbiont density and photosynthetic rates, as well as to enhance the translocation and retention of carbon within the host tissue. These findings shed new light on the interactions existing between corals and inorganic nutrients during thermal stress, and highlight the importance of phosphorus for symbiont health.

Mutualistic marine symbioses, which are widely distributed both geographically and taxonomically^1^ involve partnerships between ectothermic organisms^2^. These associations, which are thus particularly sensitive to thermal stress, are already strongly affected by climate change^3^. Among them, the coral-dinoflagellate symbioses, which dominate tropical benthic ecosystems, experience severe bleaching events in warming waters^4^.

Bleaching results from the breakdown of the symbiosis and the subsequent expulsion of the corals’ endosymbiotic dinoflagellates and/or the reduction in photosynthetic pigments^5^. However, dinoflagellates play a central role in the coral host nutrition^6^. They photosynthesize energy-rich compounds (called photosynthates), which are massively transferred to the host for its own energetic requirements and growth^6^. They also recycle the host metabolic wastes^7^ and take up dissolved inorganic nitrogen (DIN) and phosphorus (DIP) from seawater^7^,^8^,^9^. Dissolved nutrients are then transformed into essential organic molecules either respired by or stored in the symbiotic association^10^. In turn, the coral host, like all animals, is able to prey on a wide range of detrital and living particles^11^, and shares this nutrient source with its algae^12^. As most corals rely on their endosymbionts for their nutrition and survival, the increasing frequency in bleaching events may lead to a global collapse of coral reefs all around the world oceans by the 2050s^5^. There is therefore a crucial need to discern which factors, in addition to thermal stress, reduce or exacerbate the bleaching susceptibilities of scleractinian corals.

Nutrient availability is one of the key biological factors, which has to be taken into account to further explain the corals’ response to thermal stress^11^,^13^,^14^: it is indeed quite clear now that consumption of particulate organic nutrients such as plankton (i.e. heterotrophic feeding)^11^ allows corals to overcome the decrease in autotrophic nutrient acquisition by symbionts^15^ during or after thermal stress.

Therefore, species that are able to increase their heterotrophic capacities will have a higher resistance and resilience to bleaching^15^,^16^,^17^. Availability in dissolved inorganic nitrogen and phosphorus (DIN and DIP respectively) in reef environments can also strongly affect coral bleaching susceptibility^18^,^14^. However, whether these nutrients reduce or exacerbate bleaching is still subject to debate, because of the following contradicting results; on the one hand, a moderate DIN and DIP enrichment was shown to reduce bleaching and enhance coral photosynthetic performance and calcification under non-stressful^18^,^19^ and stressful conditions^13^,^20^,^21^,^22^. On the other hand, high DIN concentrations can also stimulate symbiont cell division^23^,^24^, which in turn may induce a cascading of events (such as higher retention of photosynthates in symbiont cells and CO_2_ limitation) resulting in coral bleaching^14^,^25^. Wiedenmann *et al.*^26^ finally pooled information together and proposed a conceptual model, in which the N:P ratio of inorganic nutrients in seawater was the most important factor in explaining coral bleaching. In particular, these authors showed that nitrate enrichment, coupled with phosphorus starvation resulted in an increase of sulpho- to phospholipid ratios in *Symbiodinium*, and caused a destabilization of their thylakoid membranes, increasing the corals’ bleaching susceptibility.

Significant changes in seawater N:P ratios will occur in the future, due to ocean warming and/or eutrophication of coastal waters^27^,^28^. Rising sea surface temperatures can indeed enhance near surface stratification, which will inhibit vertical mixing, and reduce access to nutrients by corals^29^. Conversely, nitrogen fixation activity of coral-associated microorganisms will increase^28^, as well as nutrient concentrations in eutrophicated coastal areas^30^. Whilst corals will not be able to change their nutritive environment, they can modulate their interaction with it.

Yet, little is known about the corals’ ability to regulate DIN and DIP uptake rates under thermal stress. In particular, no study has explored the corals’ capacity to decrease their nitrate and increase their phosphorus uptake rates under thermal stress, to keep a balanced N:P ratio inside the holobiont tissue^26^.

The first aim of this study was therefore to test whether thermal stress affects nitrogen and phosphorus uptake in different coral-symbiont (holobiont) associations, representative of common coral species in tropical reefs worldwide. For this purpose, we used a set of aquaria either maintained at 25 °C or 30 °C, and we measured the thermally induced variations in DIN and DIP uptake rates by four scleractinian coral species (*Turbinaria reniformis*, *Pocillopora damicornis*, *Pavona cactus* and *Galaxea fascicularis*) in symbiosis with clade C1 of *Symbiodinium*, and one soft coral *Heteroxenia fuscescens*, in symbiosis with clade D1. We tested the hypotheses that (i) nitrogen and phosphorus uptake rates will vary among the different holobionts under normal growth conditions; (ii) the rhythmic pulsations of *H. fuscescens*, which enhance photosynthesis^31^, will also stimulate nutrient uptake rates^32^, (iii) thermal stress will decrease nitrogen and increase phosphate uptake rates, to avoid phosphorus limitation, which may exacerbate bleaching^26^. The second aim of this study was to assess the importance of phosphorus availability for the coral resistance to thermal stress. For this purpose, we exposed one of the above species (*Pocillopora damicornis*) to a 2 μM DIP enrichment and compared its physiology and stress resistance to non-enriched corals. We tested the hypothesis that phosphorus enrichment will improve the resistance of corals to thermal stress. Broadly, our results provide detailed insights into the changes in the acquisition of inorganic nutrients by scleractinian and soft coral species and further confirm the relevance of studying shifts in N:P ratios to assess the response of reef corals to future climate change.

## Results

### First experiment

#### Physiological parameters

**Figure 1:**
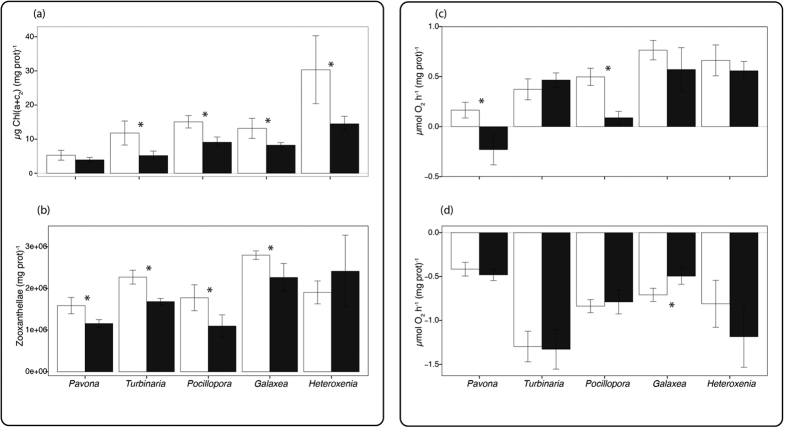
(**a**) Total chlorophyll content (μg chl (a + c_2_) (mg prot)^−1^) and (**b**) Zooxanthellae density (nb Zoox (mg prot)^−1^) of the five different coral species at 25 °C (white color) and 30 °C (black color), (**c**) Rates of net photosynthesis (μmol O_2_ h^−1^ (mg prot)^−1^) and (**d**) Respiration (μmol O_2_ h^−1^ (mg prot)^−1^) for the different coral species at 25 °C and 30 °C. Data are expressed by means ± standard deviations. Significant effects of the temperature factor were highlighted by an asterisk (*).

Significant differences were observed between species and temperature for symbiont density and chlorophyll content per mg protein (ANOVA, p = 0.008 and p = 0.0106, Fig. 1a,b). At 25 °C, *H. fuscescens* presented the highest concentration of chlorophyll (Tukey HSD, p < 0.04, Fig. 1a), while *G. fascicularis* had the highest symbiont density (Tukey HSD, p < 0.05). At 30 °C, all coral species experienced a decrease in either symbiont density or chlorophyll content. Chlorophyll concentrations indeed decreased for all species compared to 25 °C (ANOVA, p < 0.0001) except for *P. cactus* (Tukey HSD, p = 0.11). Symbiont density was significantly reduced compared to control conditions in *P. cactus*, *P. damicornis* and *T. reniformis* (Tukey HSD, p < 0.03). A significant effect of species and temperature was observed for the rates of respiration (R), net photosynthesis (P_n_), and gross photosynthesis (P_g_) normalized to protein content (ANOVA, p_1_ < 0.0001; p_2_ = 0.005, p_3_ < 0.0001, Fig. 1c,d). Both at 25 °C and 30 °C, *P. cactus* showed the lowest rate of P_n_ and R (and thus P_g_) (Tukey HSD, p < 0.03 for Pn, and p < 0.001 for R), while *T. reniformis* showed higher respiration rates compared to the other species (Tukey HSD, p < 0.008) except *H. fuscescens* (Tukey HSD, p > 0.05). At 30 °C, *P. cactus* and *P. damicornis* showed a significant reduction in P_n_ compared to 25 °C (Tukey HSD, p = 0.002 and p = 0.006 respectively).

#### Uptake of inorganic nutrients at 25 °C and 30 °C

**Figure 2:**
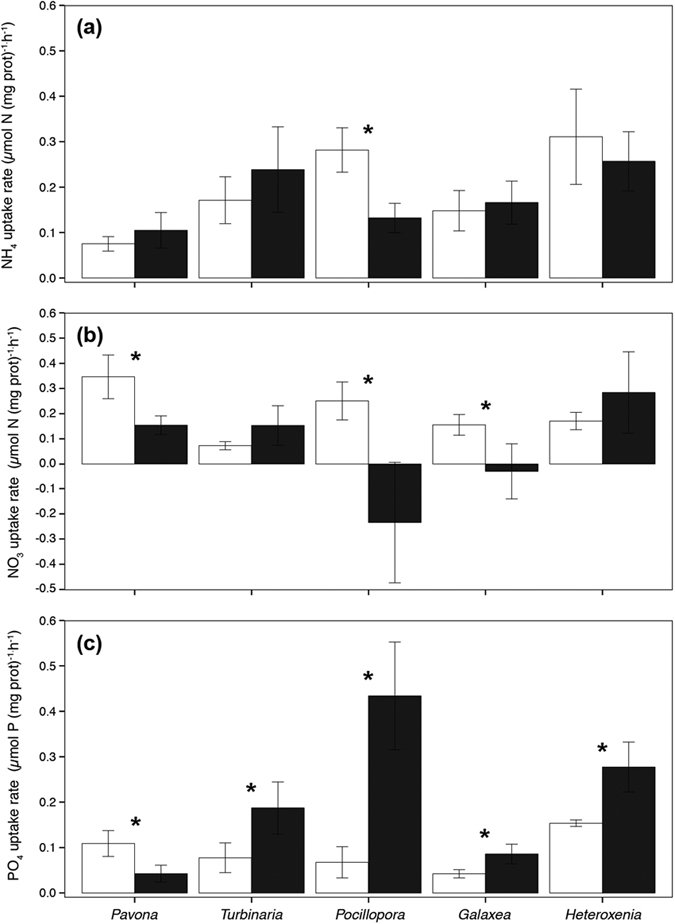
(**a**) Uptake rates of ammonium (μmol NH_4_ h^−1^ (mg prot)^−1^) (**b**) nitrate (μmol NO_3_ h^−1^ (mg prot)^−1^) and (**c**) phosphorus (μmol PO_4_ h^−1^ (mg prot)^−1^) for the different species at 25 °C (white color) and 30 °C (black color). Data are expressed by means ± standard deviations. Significant effects of the temperature factor were highlighted by an asterisk (*).

**Figure 3:**
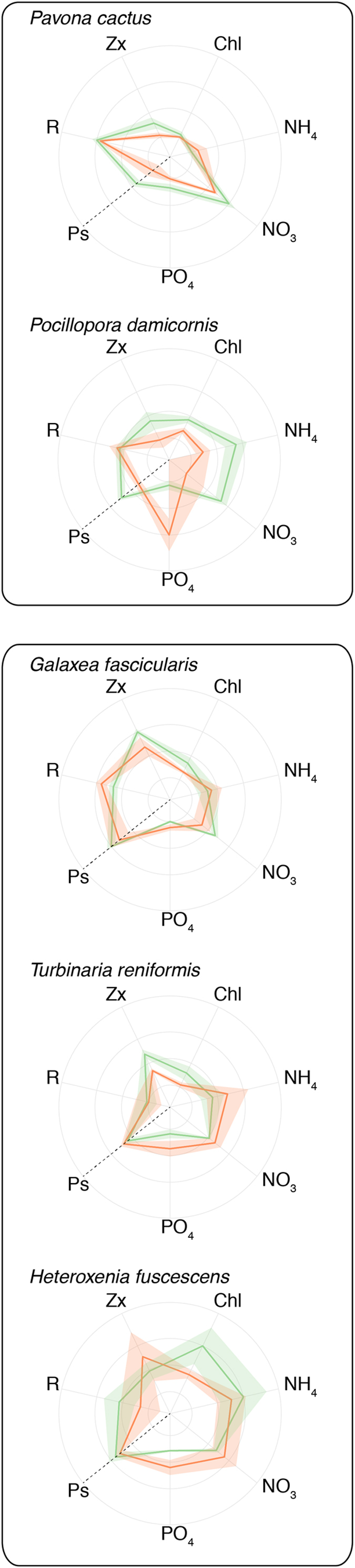
Radar plots highlighting the behavior of each coral species according to the different temperature steps (25 °C, green color and 30 °C, red color) and the studied parameters: Rates of NH_4_, NO_3_, PO_4_ uptake, chlorophyll and zooxanthellae concentrations as well as net photosynthesis and respiration rates (μmol O_2_ h^−1^ (mg prot)^−1^).

Species and temperature significantly affected nutrient uptake rates normalized to protein content (ANOVA, p = 0.008, Figs 2 and 3), or to symbiont density (ANOVA, p < 0.039, see Supplementary Table S2).

At 25 °C, *P. cactus* presented one of the highest NO_3_ uptake rate per protein content (Tukey HSD, p < 0.03, Figs 2b and 3), and one of the highest PO_4_ uptake rate per symbiont cell (Tukey HSD, p < 0.014, Supplementary Table S2). Ammonium uptake rate per symbiont cell was higher for *P. damicornis* compared to *P. cactus*, *G. fascicularis* and *H. fuscescens. P. damicornis* presented higher N-uptake:P-uptake ratios, around 9, compared to *T. reniformis and H. fuscescens* (Tukey HSD, p < 0.004, Supplementary Table S2).

Thermal stress had a significant effect on nutrient uptake rates, normalized to protein. At 30 °C, NH_4_ uptake rates significantly decreased in *P. damicornis* (Tukey HSD, p < 0.03, Fig. 2a), while NO_3_ uptake rates decreased in *P. damicornis*, *P. cactus* and *G. fascicularis* (Tukey HSD, p < 0.02). Effects of thermal stress on nitrogen uptake rates normalized to symbiont cell were significant in the presence of nitrate, with a net observed decrease in the acquisition rate of NO_3_ in *P. cactus* (Supplementary Table S8, Tukey HSD, p < 0.05) and a further but not significant decrease for *T. reniformis and G. fascicularis* (Tukey HSD, p > 0.05). PO_4_ uptake rates significantly increased in *T. reniformis, G. fascicularis*, *H. fuscescens* and *P. damicornis* when normalized to protein content (Tukey HSD, p < 0.04, Figs 2c and 3), and in the first three species when normalized to symbiont cell (Tukey HSD, p < 0.01, see Supplementary Table S2). PO_4_ uptake rates however significantly decreased for *P. cactus* (Tukey HSD, p < 0.05). Regarding the effect of tentacle pulsation of the soft coral *H. fuscescens* on nutrient uptake, pulsation rates did not vary significantly between 25 °C and 30 °C (10 puls/min at 25 °C to 8.5 puls/min at 30 °C; T-test, p > 0.05). However, nitrogen uptake rates significantly decreased as tentacles stopped pulsating (ANOVA, pNH_4_ = 0.009; pNO_3_ = 0.033, see Supplementary Table S4). A similar but non-significant trend was observed for the PO_4_ uptake rate (ANOVA, pPO_4_ > 0.05). *P. damicornis* and *G. fascicularis* showed a drastic decrease in their N-uptake:P-uptake ratios (Tukey HSD, p < 0.0074, Supplementary Fig. S3).

#### 2^nd^ Experiment: Effect of thermal stress and phosphate enrichment on the rates of carbon assimilation and translocation

**Figure 4:**
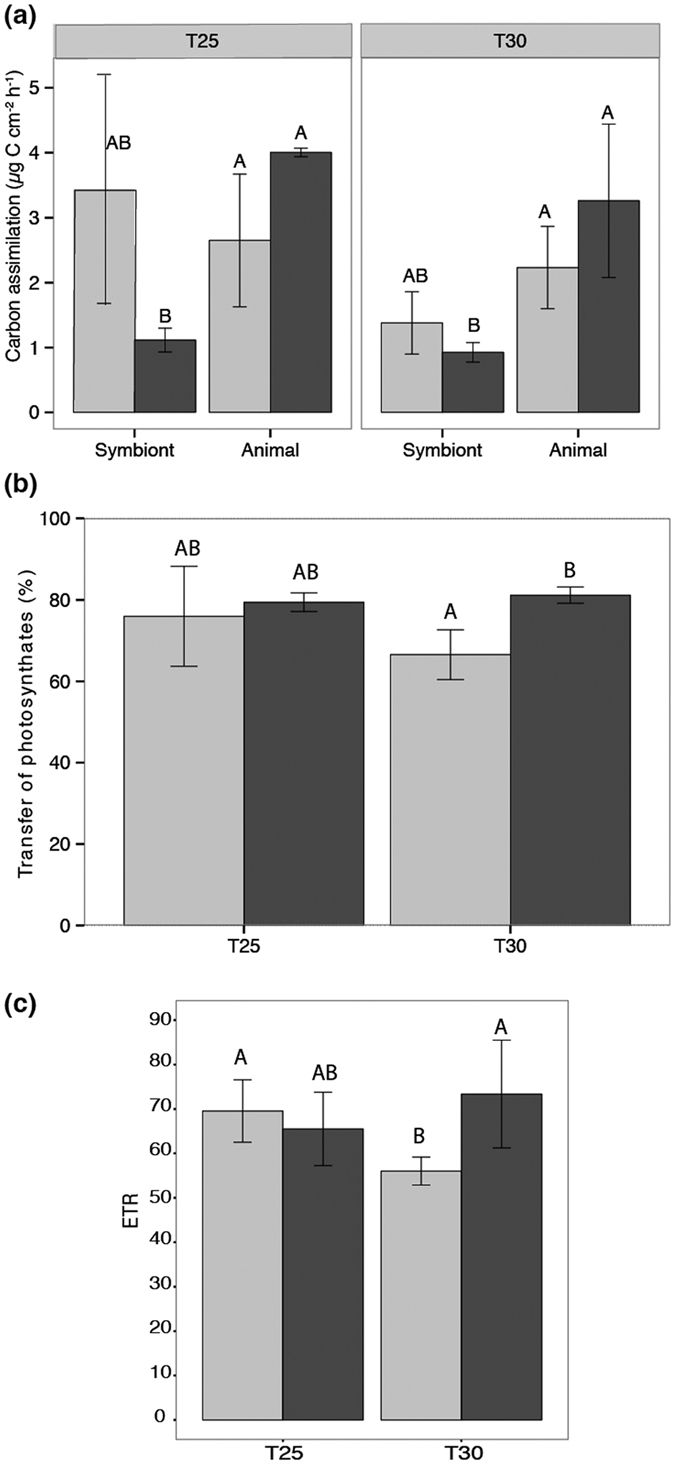
(**a**) Carbon assimilated (μg C h^−1^ cm^−2^) either in the symbiont or animal tissue fractions, (**b**) Percentage of carbon transferred from the symbiont to the animal tissue and (**c**) Maximum relative electron transport rate (rETRmax) according to the different temperature steps and the nutrient treatments (Control corals: light grey, Enriched corals: dark grey).

At 25 °C, no significant difference was observed between control and P-enriched corals regarding the symbiont density, the rETR max and NPQmax, and the gross photosynthesis expressed in equivalent carbon (P_gC_) (Tukey HSD, p > 0.05). After one week at 30 °C, control corals showed a significant decrease in symbiont density per surface area (−20%, Tukey HSD, p = 0.008, Supplementary Fig. S6), in the rates of P_gC_ (Tukey HSD, p = 0.049, Supplementary Fig. S5) and NPQmax (Tukey HSD, p = 0.048, from 0.36 to 0.25). Conversely, corals maintained under PO_4_^3−^ enrichment did not show any significant sign of bleaching nor change in their rates of gross photosynthesis compared to those at 25 °C (Tukey HSD, p > 0.05). They maintained high values of rETRmax (74 against 55 in control corals) and NPQmax (0.33 against 0.25 in control nubbins) with increasing temperature, higher than control corals (Tukey HSD, prETR = 0.023; pNPQ = 0.021, Fig. 4c). In addition, at 25 °C, ^13^C-bicarbonate labelling showed significant interactions between compartments (either zooxanthellae or animal tissue) and treatment for the rate of carbon assimilation (ANOVA, p < 0.0001). While carbon was equally incorporated in symbiont and animal tissue in control corals (Tukey HSD, p > 0.05, Fig. 4a), tissues from corals maintained under phosphorus enrichment showed a higher carbon incorporation rate and percentage (50% against 14%) (Tukey HSD, p < 0.001). The same trend was observed at 30 °C (34% against 12%; Tukey HSD, p < 0.001). Carbon translocation rates were also higher in P-enriched corals at 30 °C compared with control corals (81% against 67%; Tukey HSD p = 0.013, Fig. 4b). Symbiont respiration rates were two times higher at 25 °C and 30 °C for control corals compared to enriched nubbins, with values of 0.5 μg C cm^−2^ h^−1^ against 1–1.3 μg C cm^−2^ h^−1^ for control (Tukey HSD, p25 °C = 0.017; p30 °C = 0.0014), while the percentage of carbon respired was higher in enriched corals than control nubbins at 30 °C (44% against 25%; Tukey HSD, p < 0.05).

Finally, no significant differences were observed for the calcification rates either at 25 °C and 30 °C, with equivalent values from 14 to 16 μg C cm^−2^ h^−1^ at 25 °C and from 11 to 13 μg C cm^−2^ h^−1^ at 30 °C for control and enriched nubbins (ANOVA, p > 0.05).

## Discussion

Our analysis of the DIN and DIP uptake rates of four scleractinian and one soft tropical coral species shows that coral holobionts regulate the uptake of nutrients and points to higher DIP uptake rates under thermal stress. In addition, corals maintained under DIP enrichment avoided bleaching and maintained high rates of photosynthesis, conversely to control, non-enriched corals, which experienced both a decrease in symbiont density and in the acquisition of inorganic carbon. All together, these observations strongly suggest that phosphorus is an essential nutrient for the symbiosis during stress events. Conversely, nitrogen uptake rates significantly decreased under thermal stress, either due to coral bleaching and/or to avoid excess nitrogen in the coral tissue. Our results highlight the importance of nitrogen and phosphorus availability in the coral response to thermal stress.

Under control conditions, DIP and DIN uptake rates normalized to symbiont cell or holobiont biomass, as well as the ratios of DIN:DIP uptake, presented a threefold variation among the coral species tested, potentially reflecting different nutritional requirements and ecological trade-offs in the allocation of nitrogen and phosphorus amongst macromolecules associated with diverse functions^33^. Furthermore, we show that *H. fuscescens* exhibited higher DIN uptake rates during its pulsating activity, as previously demonstrated for the jellyfish *Cassiopeia* sp^34^. Therefore, rhythmic pulsations not only improve *H. fuscescens* photosynthesis by preventing seawater re-filtration^31^, but also enhanced its acquisition of essential nutrients.

All scleractinian coral species bleached in response to thermal stress with a reduction in rates of net photosynthesis for *P. cactus* and *P. damicornis*.

Despite this loss of symbionts and/or chlorophyll, rates of phosphate acquisition per protein content or symbiont cell were significantly enhanced in four out of the five species investigated, and for both clades C1 and D1. Since the same trend was observed with *S. pistillata* in symbiosis with another *Symbiodinium* clade (clade A)^35^, increased phosphate uptake seems to be a general response of corals to thermal stress, independently of the clade genotype or on the polyp activity. Although it might only be due to a temperature dependency of the phosphorus transporters at the cell membranes^36^, our results tend to confirm the fact that phosphorus is actively needed in corals to offset the negative effect of thermal stress^26^ and that corals underpin an active control mechanism for phosphorus uptake and allocation, as observed in plants^37^. Indeed, our second experiment, in which DIP enrichment of the seawater prevented bleaching and increased the photosynthetic efficiency and the translocation of photosynthates during thermal stress, highlights the importance of phosphorus to maintain the physiological functions in corals under thermal stress. Wiedenmann *et al.*^26^ also demonstrated that limited phosphorus availability induces a shift from phospholipids to sulpholipids in symbiont membranes, and increases the susceptibility of corals to temperature-induced bleaching^38^. In addition to being a structural membrane component, phosphorus takes part in the synthesis of nucleic acids and other energy generation processes^39^. It is also involved in cellular signalling via thylakoid protein phosphorylation^40^. It has to be noticed that although corals are able to increase their rates of phosphorus acquisition under warming conditions, a minimal concentration of inorganic phosphorus, which remains to be determined, is required to avoid bleaching.

In contrast to phosphorus, three coral species (*P. cactus*, *P. damicornis* and *G. fascicularis)* out of the five investigated showed a significant decrease in nitrogen uptake rates normalized to protein content. Nitrogen uptake rates normalized to symbiont cell, however, remained unchanged between 25 °C and 30 °C, except for nitrate in *P. cactus.*

A similar finding was observed with the scleractinian coral *S. pistillata* associated with clade A1 symbionts^35^, which significantly decreased both nitrate and ammonium uptake rates down to zero, at 33 °C. These results suggest that the reduction in nitrogen uptake is a general response of corals to thermal stress, independent of the clade genotype. Whether this reduction is a consequence of bleaching (and reduced symbiont activity), or is due to a physiological control or feedback aimed at decreasing the amount of nitrogen entering the symbiotic association, requires further investigation. Both hypotheses have pros and cons. The first hypothesis is in agreement with studies showing that nitrogen uptake is mostly driven by the symbionts^8^,^9^,^41^,^42^. In *H. fuscescens*, which did not experience any bleaching or decrease in photosynthetic activity, due to its pulsating activity and/or its association with the thermally resistant clade D1^43^,^44^,^45^, no decrease in nitrogen uptake rate was indeed observed. A decrease in nitrogen uptake rates in the scleractinian coral species may lead to nitrogen limitation, which in turn may impair protein repair^23^, weaken the photosynthetic capacities, and decrease carbon fixation, as already noted in mulberry leafs^46^. This may explain why a supplementation of seawater with ammonium or nitrate was shown to increase the resilience of coral species to stress-induced bleaching^21^,^22^,^47^. On the other hand, the second hypothesis is in agreement with studies showing an increased bleaching susceptibility of heat-stressed corals in presence of high nitrogen and low phosphorus concentrations^26^,^48^,^49^,^50^. High nitrogen availability for symbionts during thermal stress tends to favour symbiont growth and reduce the amount of photosynthates transferred to the host^14^, together with inducing a higher phosphorus limitation^26^. This could explain the reduction in nitrogen uptake rates by the symbiotic association during thermal stress, in order to reduce the amount of nitrogen available to symbionts.

Overall, the decrease in the N:P uptake ratios during thermal stress, which is consistent for most coral species, may imply that the corals internally experience an increase in their N:P ratio, maybe due to reallocation of nitrogen that was stored in tissue reserves^10^. The relevance of the N:P ratio for terrestrial and marine symbiotic associations has recently been discussed through a meta-analysis^50^ which showed that a decoupling in mutualism performance occurs whenever phototrophs benefit from nutrient enrichment at the expense of their heterotrophic partners. It has also been demonstrated in recent studies^26^,^51^, in which changes in seawater nutrient ratios induced changes in carbon acquisition, translocation and allocation within the symbiosis. The benefits that the symbiotic association retrieves from a low N:P ratio condition are highlighted in our second experiment, in which the supplementation of seawater with 2 μM phosphate, and the subsequent decrease in the N:P ratio, prevented bleaching, increased the photosynthetic capacities, the carbon translocation, as well as the carbon retained into animal biomass during thermal stress.

Understanding how corals respond to alterations in seawater N:P ratio, or how they adjust their internal N:P ratio in response to environmental changes, is an important task for coral reef research today. Our results clearly show that corals are phosphorus-limited during thermal stress and that they are able to increase their acquisition of phosphorus independently of the *Symbiodinium* clade involved in the symbiotic association. Further investigations, which will trace the fate of nitrogen and phosphorus within the symbiosis at elevated temperatures, are however needed to bring detailed insights into the processes involved in the nutrient acquisition and allocation by corals under thermal stress.

## Materials and Methods

### First experiment: experimental design and biological material

The first experiment was conducted with four tropical scleractinian coral species, belonging to different families, *Turbinaria reniformis* (Dendrophylliidae), *Pocillopora damicornis* (Pocilloporidae), *Pavona cactus* (Agariciidae), *Galaxea fascicularis* (Oculinidae) and the soft coral species *Heteroxenia fuscescens* (Xeniidae), all originating from the Red Sea. These corals species were selected for their different morphology types and because they are good representatives of common coral families in tropical reefs worldwide^52^,^53^. Coral colonies were maintained in aquaria for several months at 25 °C ± 0.5. The total daily light received by our corals in these conditions (13 E m^−2^ d^−1^ which corresponds to 150 μmol photons m^−2^ s^−1^) was equivalent to the one received on reefs at 3–5 m depth (a typical ideal day with no cloud generates a total of 14 E m^−2^d^−1^ day at 3 m depth, while a cloudy day generated 6.2 E m^−2^d^−1^ 54. A total of 48 nubbins per species was prepared by cutting 4 parental colonies (12 nubbins per colony) with a bone cutter for hard corals or scissors for soft corals. Nubbins were then evenly divided in twelve 20 L tanks (with 4 nubbins per species per tank) in order to maximize the genetic diversity. Aquaria received a continuous supply of oligotrophic seawater (<0.5 μM dissolved inorganic nitrogen and <0.1 μM inorganic phosphorus) at a flow rate of 20 L h^−1^ (i.e. one tank renewal every hour).

Control conditions were maintained using submersible resistance heaters (Visi-ThermH Deluxe, Aquarium Systems, France) and metal halide lamps (Philips, HPIT 400 W, Distrilamp, Bossee, France). Submersible pumps ensured proper water mixing inside each tank. Nubbins were fed twice a week for 2 weeks with *Artemia salina* nauplii to improve their recovery. Three weeks prior to the experiments, feeding was stopped to minimize the impact of organic nutrients on the uptake rates of inorganic nutrients (described below).

Before the measurements, four nubbins from each species (one per colony) were frozen at −80 °C for the *Symbiodinium* clade determination. DNA was extracted using the DNeasy Plant mini Kit (Qiagen) for *H. fuscescens* and the PowerSoil DNA Isolation Kit (MO-BIO) for the other species. For the determination of symbiont phylogeny, we followed the protocol of Santos *et al.*^55^, and sequenced a 600 to 700 bps fragment of chloroplastic 23S ribosomal gene. All coral species harboured *Symbiodinium* clade C1, with the exception of *H. fuscescens*, which hosted clade D1 (see S1).

### Uptake of inorganic nutrients at 25 °C and 30 °C

Six aquaria were kept under control conditions. In the remaining six tanks, seawater temperatures were raised from 25 °C ± 0.5 °C (time T_0_ −25 °C) to 30 °C ± 0.5 °C over a 10 day-period (0.5 °C per day) and maintained constant for 10 days (time T_1_ −30 °C). This thermal stress was chosen according to the highest temperature monitored in the northern Red Sea^56^. Samples from the 5 coral species were collected from both the control and experimental tanks at T_0_ and T_1_ to assess their photosynthetic performances as well as their uptake rates of DIN and DIP (NH_4_, NO_3_, PO_4_) as described below. Nubbins were then frozen at −20 °C prior to the determination of the chlorophyll and protein content as well as the symbiont density. Throughout the experiment, inorganic nutrient concentrations were measured in the different aquaria twice a week using an Autoanalyzer (Alliance Instrument, AMS, France), following the protocol of Le Corre & Tréguer^57^. Concentrations remained below 0.5 μM for total DIN and below 0.1 μM for DIP.

Net photosynthesis (P_n_) and respiration (R) rates were assessed at 0 and 150 μmol photons m^−2^ s^−1^ on four coral nubbins per species (from 4 different colonies) and temperature using the respirometry technique^58^.

Gross photosynthesis (Pg) was assessed by adding the absolute value of R to Pn. Data were normalized to total protein content and surface area for hard coral species. For protein and chlorophyll concentrations (a and c_2_) as well as symbiont density, tissue was removed from the skeleton in filtered seawater (FSW) using an air-brush, homogenized using a potter grinder and divided into three aliquots for each measurement, performed according to Rodolfo-Metalpa *et al.*^59^ for symbionts, Jeffrey & Humphrey^60^ for chl a and c_2_ and Smith *et al.*^61^ using a BCAssay kit (Interchim, Montluçon, France) for protein content determination. Surface area of nubbins were assessed using the wax dipping technic^62^.

Inorganic nutrient uptake was assessed, at the same time for the control and experimental tanks, at T_0_ and T_1_, by the depletion technique^63^. In short, for each nutrient and incubation time, four nubbins per species (one nubbin per colony) were placed in individual 200-mL beakers maintained in a water bath at the respective experimental temperatures (25 °C or 30 °C) and light intensity (150 μmol photons m^−2^ s^−1^). Three control beakers, without coral nubbin, were used to monitor for potential changes in water chemistry due to adsorption onto beaker surface, consumption by microbial activity, or to air contamination. After a 30 min acclimation period for corals, seawater was enriched with a stock solution of either 10 mM ammonium chloride (NH_4_Cl), Sodium nitrate (NaNO_3_), or sodium dihydrogen phosphate (NaH_2_PO_4_), in order to reach a final concentration of 3 μM for all nutrients. Stirring bars were added to ensure a proper homogenization of the nutrient mixture in the beakers. Water samples (10 mL) were collected from each beaker and filtered through a 45 micron syringe filter immediately after the enrichment and then subsequently after 15 min, 30 min, 45 min, 60 min and 90 min. Ammonium concentrations were determined immediately after sampling, according to the spectrofluorimetric method of Holmes *et al.*^64^. Samples for nitrate and phosphate were frozen, prior to measurements using an autoanalyzer (Alliance Instrument, AMS, France).

Uptake rates of each inorganic nutrient were calculated as the difference in concentration between the 0 and 90 minutes time points, after data correction for the diminution of the beakers’ volume and verification of the linearity in the depletion during the experiment. Uptake rates were normalized to the symbiont density (which changed during the thermal stress) or to the total protein content, which did not change during the whole experiment. N-uptake:P-uptake ratios were calculated at T_0_ and T_1_ by dividing the total amount of nitrogen (NH_4_ and NO_3_) by the amount of phosphorus taken up.

### Effect of soft coral pulsation on the uptake rates of inorganic nutrients

Polyp pulsation by *H. fuscescens* under non stressful conditions was shown to improve photosynthesis, by enhancing oxygen and inorganic carbon exchange between seawater and polyp tissue^31^ and was suggested to favor uptake of inorganic nutrients^32^. To investigate whether thermal stress had an effect on the rhythmic pulsation of this species, we monitored the number of tentacle pulsation per minute of four nubbins of *H. fuscescens* maintained under non-stressful (25 °C) and thermal stress (30 °C) conditions. In addition, we also assessed the effect of polyp pulsation on the uptake rates of inorganic nutrients (NH_4_, NO_3_ and PO_4_). For this purpose, additional 32 coral nubbins (8 nubbins per colony) were cut from 4 parental colonies, different from the ones used for the uptake rates assessment. A clove oil solution (Phytosun aroms; Omega Pharma, France) was chosen to anaesthetize corals and inhibit their movements. For this experiment, each coral nubbin was only maintained for 1 h30 under a diluted clove oil solution (0.2 ppt). A concentration lower than 0.5 ppt was shown to be completely harmless to corals^65^,^66^. In addition, preliminary tests were performed to test the harmlessness of the clove oil at different dilutions, by comparing rates of photosynthesis and respiration as well as chlorophyll and symbionts concentrations with and without clove oil addition.

We didn’t observe any effect of clove oil on the above parameters for concentrations below 0.5 ppt (results not shown). Following optimization, eight nubbins per nutrient were disposed in distinct 200-mL beakers, half of them were placed in FSW with clove oil and the other half in FSW only (control condition). When coral tentacles were totally immobilized by the clove oil, NH_4_, NO_3_ or PO_4_ solutions were added to reach a final concentration of 3 μM, N or P, and nutrient depletion was followed as described above. Nutrient uptake rates were normalized to the total protein concentration of each nubbin.

### Second experiment: Carbon budget under PO_4_^3−^ supply

Based on the results obtained in the first experiment, we tested the effects of phosphate enrichment and thermal stress on the carbon budget of *Pocillopora damicornis*, which was chosen as a model species. We used ^13^C-bicarbonate, to follow the inorganic carbon incorporation rate and transfer between the host and symbionts, as described in Tremblay *et al.*^67^. 160 nubbins were generated from four parental colonies and equally distributed among 8 aquaria maintained under control conditions as previously described. Corals were exposed to: 1) natural seawater, called control conditions, with low nutrient levels (0.5 μM N and 0.1 μM P; 4 aquaria) and 2) phosphorus enriched conditions, called “P” (0.5 μM N and 2 μM P; 4 aquaria). Tanks were continuously supplied with a solution of NaH_2_PO_4_ pumped from a stock solution via a peristaltic pump. The stock solution was renewed every three days and added to the tanks at a constant flow rate of 0.3 L h^−1^.

Nutrient concentrations in the tanks were monitored twice a week using an Autoanalyzer (Alliance Instrument, AMS, France). Nubbins were maintained under these conditions at 25 °C (T_25_) for four weeks. Seawater temperatures were then raised from 25 °C ± 0.5 °C to 30 °C ± 0.5 °C over a 10 day-period in half of the tanks and was kept constant for 7 days (T_30_).

Rates of net photosynthesis (Pn), R and P_g_, as well as zooxanthellae density, were measured as previously described at 0 and 150 μmol photons m^−2^ s^−1^ on six nubbins per treatment at T_25_ and T_30_. Symbiont respiration rates were assessed on six freshly isolated symbiont samples according to Tremblay *et al.*^67^. The resulting oxygen fluxes were converted to carbon equivalent based on molar weights according to Anthony & Fabricius^68^. The maximum relative electron transport rate (rETRmax) and the non-photochemical quenching (NPQmax) were assessed on the same six coral nubbins, at 25 °C and 30 °C at different light levels using a Pulse Amplitude Modulation (PAM) fluorometer (Walz, Germany) according to Ralph and Gademann^69^. Calcification rates were measured on six nubbins per treatment using the buoyant weight technique^70^. All results were normalized to the surface area of each respective nubbin.

The carbon budget was assessed at the end of T_25_ and T_30_ using the H^13^CO_3_ labelling technique according to Tremblay *et al.*^67^. For each treatment, eight nubbins (2 per colony) were disposed in individual beakers filled with 200 mL FSW, enriched with a final concentration of 0.6 mM of NaH^13^CO_3_ (98 atom %^13^C, no. 372382, Sigma-Aldrich, St Louis, MO, USA). Nubbins were incubated for 5 h and transferred to beakers containing non-enriched seawater (FSW) for two chase periods (0 and 24 h). At the end of each chase period, 4 nubbins were directly frozen at −20 °C to assess ^13^C enrichment in animal tissue and symbionts. Additionally, four control nubbins were incubated in non ^13^C- enriched seawater. The resulting %^13^C enrichment was quantified using a Delta Plus Mass spectrometer coupled to a C/N analyser (Thermofisher Scientific, Bremen, Germany).

### Statistical analyses

Statistical analyses were performed using Statistica 10. Data were checked for normality using the Kolmogorov-Smirnov test with Lilliefors correction and for Homoscedasticity using Levene’s test. Data were log-transformed if necessary. Two ways ANOVAs were used to test (i) the effect of temperature and coral species (independent factors) on nutrient uptake rates, and other physiological parameters measured in the first experiment; (ii) the effect of temperature and phosphorus enrichment (independent factors) on all parameters measured in the second experiment. Calcification rates and the maximum rETR and NPQ were analysed using a general linear model for parametric repeated measures analysis of variance with “Temperature” as dependent variable and “Treatment” as categorical predictor. In case the assumption of sphericity (independency of the repeated measures) was not fulfilled, the hypotheses were tested using the multivariate approach (Wilks test) for repeated measurements. Differences in the rates of carbon assimilation and the percentage of carbon remaining within the symbiosis, were assessed using three-ways ANOVA (with temperature, phosphorus enrichment and host-symbiont compartments as independent factors). A t-test was finally processed to test the effect of clove oil on nutrient uptake rates by *H. fuscescens*. In case significant differences were observed between treatments, analyses were followed by a posteriori test (Tukey’s test). Differences were considered significant when p < 0.05.

## Additional Information

**How to cite this article**: Ezzat, L. *et al.* Limited phosphorus availability is the Achilles heel of tropical reef corals in a warming ocean. *Sci. Rep.* **6**, 31768; doi: 10.1038/srep31768 (2016).

## Supplementary Material

Supplementary Information
