# Supplementary material for: Limited phosphorus availability is the Achilles heel of tropical reef corals in a warming ocean
Source: Sci Rep. 2016 Aug 17;6:31768. doi: 10.1038/srep31768 (PMC4987665; doi:10.1038/srep31768)
Supplement: Supplementary Information [file srep31768-s1.pdf]

Supplementary Information

Title: Limited phosphorus availability is the Achilles' heel of tropical reef corals in a warming ocean

Authors :

Leïla Ezzat

Jean-François Maguer

Renaud Grover and

Christine Ferrier-Pagès

Supplementary Table S1. Determination of Symbiodinium clade via BLASTn

Chloroplast Partial 23S rRNA gene	Host organism	Identity (%)	Site (bp)	E value
***Symbiodinium* Clade C1**	*P.cactus*	100	623	0.0
	T.reniformis	99	599	0.0
	P.damicornis	99	623	0.0
	G.fascicularis	99	621	0.0
***Symbiodinium* Clade D1**	*H.fuscescens*	99	718	0.0

Supplementary Table S2. Nutrient uptakes normalized by zooxanthellae density

The uptake of nutrients (NH_4, NO_3 and PO_4; µmol of Nutrient (Nb Zoox)$^{-1}$ h^{-1}) normalized by the density of zooxanthellae according to the different coral species and the temperature steps (25°C and 30°C). Significant differences due to temperature were highlighted by an asterisk(*)

Species	Temperature	NH_4 uptake	STDEV	NO_3 uptake	STDEV	PO_4 uptake	STDEV
P.cactus	T25	2.84E-08	1.58E-08	*2.23E-07	8.53E-08	7.1E-08	3.54E-08
	T30	5.19E-08	1.68E-08	1.04E-07	2.07E-08	4.93E-08	1.56E-08
H.fuscescens	T25	4.45E-08	1.77E-08	1.24E-07	1.76E-07	*2.9E-08	5.72E-09
	T30	1.21E-07	5.06E-08	1.22E-07	4.48E-08	1.32E-07	7.57E-08
T.reniformis	T25	8.99E-08	4.88E-08	3.04E-07	1.93E-07	*1.01E-08	5.02E-09
	T30	8.72E-08	2.44E-08	9.01E-08	3.36E-08	7.1E-08	1.85E-08
P.damicornis	T25	1.09E-07	3.04E-08	9.66E-08	2.25E-08	*3.65E-08	9.39E-09
	T30	1.05E-07	3.69E-08	1.52E-07	7.12E-08	1.84E-09	1.29E-09
G.fascicularis	T25	3.92E-08	2.75E-08	1.45E-07	4.84E-08	*2.01E-08	6.54E-09
	T30	4.25E-08	2.22E-08	9.71E-08	1.51E-08	4.11E-08	6.54E-09

Supplementary Figure S3. N-uptake: P-uptake

Ratio of total nitrogen (NH_4+NO_3) uptake on phosphorus uptake that have been previously normalized by µmol of N (mg prot)$^{-1}$ h^{-1}, according to the different coral species and the temperature steps. Significant effects of temperature were highlighted by an asterisk (*). Temperature steps (T25°C and T30°C) are represented by white and black bars, respectively.

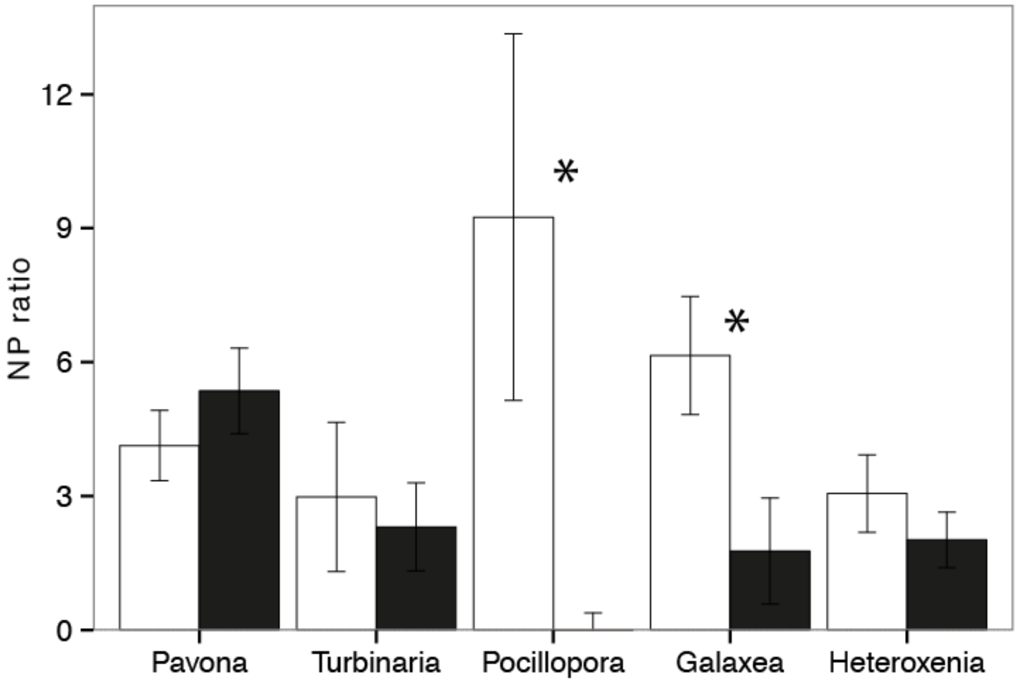

Supplementary Table S4. Uptake of inorganic nutrients by *H.fuscescens*
Uptake of inorganic nutrients (NH4, NO3, PO4)
in umol of nutrients (mg prot)-1mn-1. Significant effects of clove oil solution in decreasing polyp pulsation were highlighted by an asterisk (*).

Treatment	Mean	STDEV
NH4	0.023383	**0.006532**
NH4 EO	0.010009*	**0.001504**
NO3	0.010998	**0.00374**
NO3 EO	0.005465*	**0.001504**
PO4	0.000471	**0.000104**
PO4 EO	0.0003	**8.96E-05**

Supplementary Figure S5. Gross photosynthesis of *P.damicornis*

Rates of gross photosynthesis (µg C cm^{-2} h^{-1}) of *P.damicornis* according to the nutrient treatment and the temperature steps (25°C and 30°C) relative to the second experiment. Control and P-enriched treatments are represented by grey and dark grey bars, respectively.

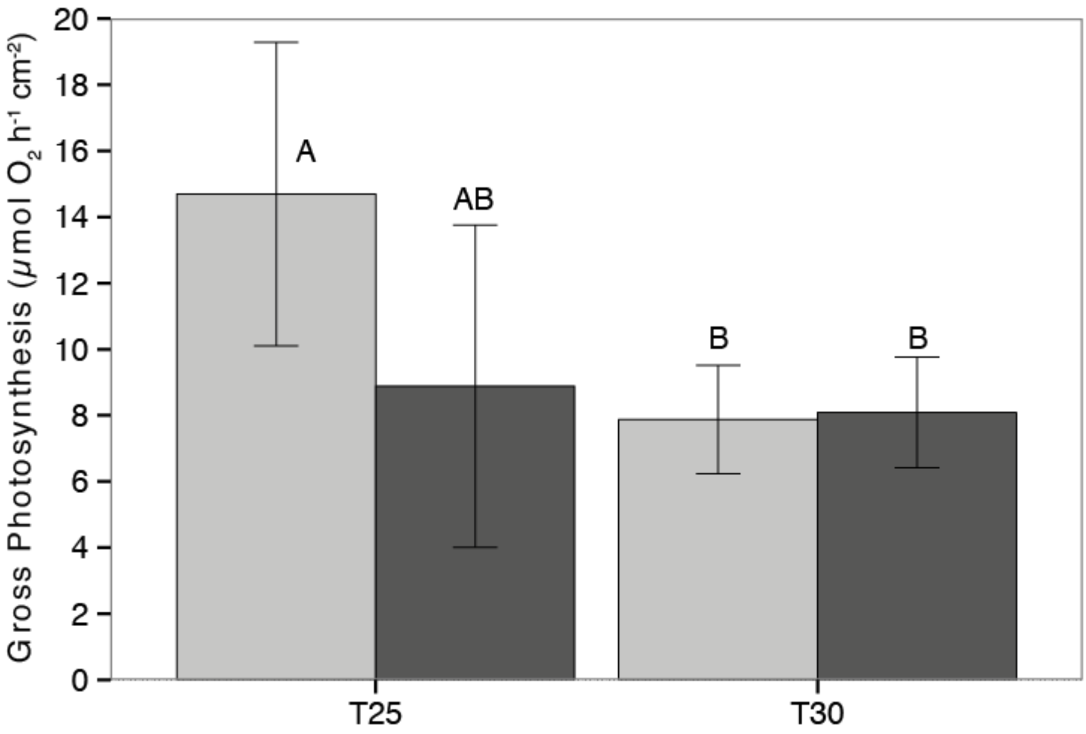

Supplementary Figure S6. Zooxanthellae density of *P.damicornis*

Density of zooxanthellae (Nb. Zooxanthellae cm^{-2}) of *P.damicornis* according to the different nutrient treatment and the temperature steps (25°C and 30°C), relative to the second experiment. Control and P-enriched treatments are represented by grey and dark grey bars, respectively.

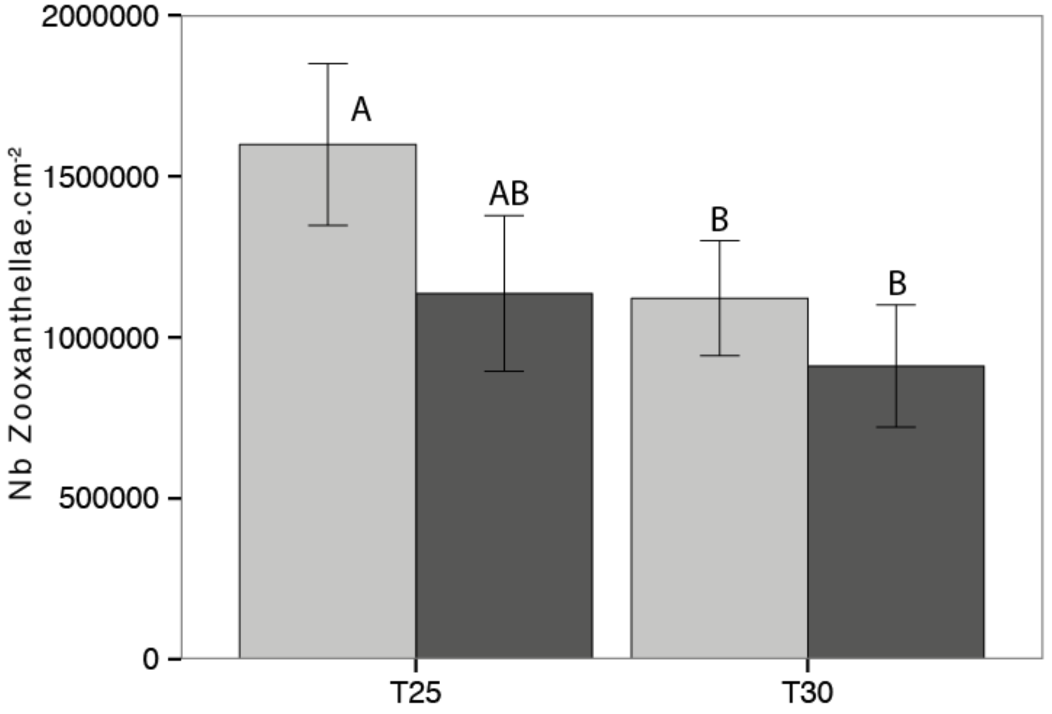

Supplementary Table 7. Summary of ANOVAs testing the effect of temperature (25°C, 30°C) and species or treatment (only) on the main physiological parameters. P values are considered significant for $p<0.05$.

Source of variation	Df	F	p
Protein content (hard corals) **(mg prot cm-2)**			
**Species**	3	19.29	**< 0.0001**
**Temperature**	1	0.11	0.746
**Species x Temperature**	3	3.13	**0.04**
Chlorophyll content **(µg Chl(a+c2)*(mg prot)-1)**			
**Species**	4	45.8	**< 0.0001**
**Temperature**	1	116.2	**< 0.0001**
**Species x Temperature**	4	2.1	**0.0106**
Zooxanthellae density **(Nb zooxanthellae mg prot)-1)**			
**Species**	4	10.76	**0.003**
**Temperature**	1	17.51	**< 0.0001**
**Species x Temperature**	4	4.29	**0.008**
Net Photosynthesis **(µmol O2 (mg prot)-1)**			
**Species**	4	54.94	**< 0.0001**
**Temperature**	1	10.6	**0.003**
**Species x Temperature**	4	8.95	**< 0.0001**
Respiration **(µmol O2 (mg prot)-1)**			
**Species**	4	34.88	**< 0.0001**
**Temperature**	1	1.71	0.201
**Species x Temperature**	4	4.54	**0.005**
Gross Photosynthesis **(µmol O2 (mg prot)-1)**			
**Species**	4	30.86	**< 0.0001**
**Temperature**	1	5.44	**0.026**
**Species x Temperature**	4	10.99	**< 0.0001**

Supplementary Table 8. Summary of ANOVAs testing the effect of temperature (25°C, 30°C) and species or treatment (only) on the main physiological parameters. P values are considered significant for p<0.05.

Source of variation	Df	F	p
Ammonium uptake rate **(µmol NH_4 h^{-1} mg $prot^{-1}$)**			
**Species**	4	12.22	**<0.0001**
**Temperature**	1	0.91	0.347
**Species x Temperature**	4	4.24	**0.008**
Nitrate uptake rate **(µmol NH_4 h^{-1} mg $prot^{-1}$)**			
**Species**	4	7.46	**<0.0001**
**Temperature**	1	15.9	**<0.0001**
**Species x Temperature**	4	10.32	**<0.0001**
Phosphorus uptake rate **(µmol PO_4 h^{-1} mg $prot^{-1}$)**			
**Species**	4	22.46	**<0.0001**
**Temperature**	1	51.38	**<0.0001**
**Species x Temperature**	4	20.07	**<0.0001**
Ammonium uptake rate **(µmol NH_4 h^{-1} Nb $Zoox^{-1}$)**			
**Species**	4	8.109	**<0.0001**
**Temperature**	1	4.201	0.05
**Species x Temperature**	4	2.270	0.083
Nitrate uptake rate **(µmol NO_3 h^{-1} Nb $Zoox^{-1}$)**			
**Species**	4	1.011	0.419
**Temperature**	1	4.702	**0.039**
**Species x Temperature**	4	2.552	0.062
Phosphorus uptake rate **(µmol PO_4 h^{-1} Nb $Zoox^{-1}$)**			
**Species**	4	5.387	**0.002**
**Temperature**	1	7.44	**0.011**
**Species x Temperature**	4	7.489	**<0.0001**
NP ratio			
**Species**	4	3.9	**0.013**
**Temperature**	1	32.31	**0.0001**
**Species x Temperature**	4	12.16	**0.0001**
Ammonium uptake rate at 25°C (H.fuscescens) **(µmol NH_4 mn^{-1} mg $prot^{-1}$)**			
**Treatment**	1	14.36	**0.009**
Nitrate uptake rate at 25°C (H.fuscescens) **(µmol NO_3 mn^{-1} mg $prot^{-1}$)**			
**Treatment**	1	7.538	**0.033**
Phosphorus uptake rate at 25°C (H.fuscescens) **(µmol PO_4 mn^{-1} mg $prot^{-1}$)**			
**Treatment**	1	2.214	0.197

Supplementary Table 9. Summary of ANOVAs testing the effect of temperature (25°C, 30°C) and nutritional treatment on the different physiological parameters for the second experiment. P values are considered significant for p<0.05.

Source of variation	Df	F	p
Gross photosynthesis **(μg C h^{-1} cm^{-2})**			
**Temperature**	1	6.845	**0.019**
**Treatment**	1	1.451	0.247
**Temperature x Treatment**	1	1.804	0.199
Zooxanthellae density **(Nb Zoox cm^{-2})**			
**Temperature**	1	6.701	**0.018**
**Treatment**	1	1.721	0.204
**Temperature x Treatment**	1	6.676	**0.018**
Symbiont respiration rate, R_s **(μg C cm^{-2} h^{-1})**			
**Temperature**	1	0.51	0.484
**Treatment**	1	31.7	**<0.0001**
**Temperature x Treatment**	1	1.23	0.282
Host Respiration rate, R_H **(%)**			
**Temperature**	1	4.15	0.057
**Treatment**	1	13.68	**0.002**
**Temperature x Treatment**	1	0.18	0.673
Calcification rate, Cc **(μg C cm^{-2} h^{-1})**			
**Temperature**	1	0.005	0.943
**Treatment**	1	1.238	0.292
**Temperature x Treatment**	1	<0.000	0.984
rETRmax			
**Temperature**	1	0.58	0.468
**Treatment**	1	9.051	0.017
**Temperature x Treatment**	1	5.589	**0.046**
NPQmax			
**Temperature**	1	26	**0.001**
**Treatment**	1	13.68	**0.002**
**Temperature x Treatment**	1	0.74	0.416

Supplementary Table S10. Summary of factorial ANOVAs testing the effect of temperature (25°C, 30°C), nutritional treatment as well as the symbiotic compartment on the rates of carbon assimilation and transfer

Carbon assimilated after 24h **(µg C $h^{-1}cm^{-2}$)**			
**Compartment**	1	43.79	**<0.0001**
**Temperature**	1	11.24	**0.002**
**Treatment**	1	1.62	0.212
**Compartment x Temperature**	1	2.18	0.15
**Compartment x Treatment**	1	27.16	**<0.0001**
**Temperature x Treatment**	1	1.6	0.216
**Compartment x Temperature x Treatment**	1	3.29	0.08
Carbon remaining within the symbiosis after 24h **(%)**			
**Compartment**	1	51.18	**<0.0001**
**Temperature**	1	0.88	0.357
**Treatment**	1	1.12	0.3
**Compartment x Temperature**	1	25.43	**<0.0001**
**Compartment x Treatment**	1	1.42	0.243
**Temperature x Treatment**	1	0.37	0.548
**Compartment x Temperature x Treatment**	1	2.37	0.135
Rate of photosynthates transferred after 24h **(µg C $h^{-1}cm^{-2}$)**			
**Temperature**	1	26.5	**<0.0001**
**Treatment**	1	2.88	0.109
**Temperature x Treatment**	1	21.58	**<0.0001**
Rate of photosynthates transferred after 24h **(%)**			
**Temperature**	1	1.604	0.225
**Treatment**	1	9.648	**0.007**
**Compartment x Treatment**	1	3.24	0.092
